# Medication Adherence and Common Barriers for Caregivers of Preschool Children with Pediatric Glaucoma

**DOI:** 10.1155/2022/6389822

**Published:** 2022-12-31

**Authors:** Zitian Liu, Yuning Zhang, Pingping Liu, Wenxin Yang, Xinyan Li, Yimin Zhong, Yangfan Yang, Xing Liu, Huiming Xiao, Minbin Yu, Wenmin Huang

**Affiliations:** State Key Laboratory of Ophthalmology, Zhongshan Ophthalmic Center, Sun Yat-sen University, Guangdong Provincial Key Laboratory of Ophthalmology and Visual Science, Guangdong Provincial Clinical Research Center for Ocular Diseases, Guangzhou, China

## Abstract

**Purpose:**

To investigate the medication adherence among caregivers of preschool children with pediatric glaucoma and to elucidate common barriers leading to poor adherence.

**Methods:**

A cross-sectional study. Caregivers of preschool children with pediatric glaucoma completed a questionnaire on demographic information of caregivers, demographic and disease characteristics of children, caregivers-reported medication adherence (by an adapted Morisky Adherence Scale), and possible 13 barriers.

**Results:**

Overall 132 questionnaires were considered valid. Thirty-six percent of all reported poor medication adherence. Caregivers' age and self-evaluated knowledge about pediatric glaucoma showed a significant difference between the adherent and nonadherent groups (*P* < 0.05). Nineteen percent of all reported only one barrier as important, 65% cited multiple barriers, and 16% cited no barriers. Anxiety and depression were cited as important by most caregivers in both groups. Univariate logistic regression analysis demonstrated that difficulty with the acquisition of medications (OR, 2.5; 95% CI, 1.1–5.7; *P*=0.025), difficulty with medication schedule (OR, 2.3; 95% CI, 1.0–5.0; *P*=0.043), and high expenses for medications (OR, 4.8; 95% CI, 1.4–15.9; *P*=0.011) were significantly associated with higher odds of poor adherence.

**Conclusions:**

Over one-third of caregivers of preschool children with pediatric glaucoma were in poor medication adherence. Nearly two-thirds of caregivers cited multiple barriers simultaneously as important hindrances to medication usage. Anxiety and depression, difficulty with the acquisition of medications, difficulty with the medication schedule, and high expenses for medications were prominent barriers. Individualized solutions should be provided according to reported barriers by each caregiver and the other most common barriers.

## 1. Introduction

Pediatric glaucoma is a variety of disorders characterized by elevated intraocular pressure (IOP) and specific optic neuropathy in infants and children [1, 2]. It not only results in a lifetime of severe visual impairment and low quality of life to children but brings significant psychosocial impacts and serious economic burdens to their families [3–6]. Lowering IOP has been proven to be effective to retard the progression of visual loss [7–9]. Although surgical treatment is always taken as a necessary method to reduce IOP, topical antiglaucoma medication is often needed for additional IOP control following surgery or as initial first-line therapy for some second-acquired childhood glaucoma [10–13].

Well adherence to antiglaucoma medication is significantly associated with good control of visual loss for glaucoma patients [14]. Poor medication adherence, however, is still a prevalent problem. Patients often have multiple and unique sets of barriers [15]. By now, there are many studies focusing on the issue of medication adherence of adults with glaucoma [15–17]. However, medication adherence of patients with pediatric glaucoma has not been given enough attention.

Unlike adults with glaucoma, children could not instill medications independently, especially those preschoolers. The inevitable resorting to help from caregivers may make medication adherence dependent more on caregivers, as that happened to children with other chronic diseases like asthma [18, 19]. Possible barriers to medication adherence of caregivers of children could be very different from those of adults, like children's resistance to medication instillation. They may also come from caregivers, like caregivers' poor health literacy and depressive emotion [18, 20]. However, what specific barriers of caregivers would be significantly associated with poor medication adherence among preschool caregivers of children with pediatric glaucoma and how far they act remained unclear.

For promoting the clinical management of pediatric glaucoma, it is indispensable to figure out medication adherence and clarify reasons for nonadherence. This study aimed to investigate the current situation of medication adherence among caregivers of preschool children with pediatric glaucoma, and from the perspective of their caregivers, to identify common barriers that lead to poor adherence.

## 2. Methods

The study was conducted following the tenets of the Declaration of Helsinki. Ethical approval was obtained from the Ethics Committee of the Zhongshan Ophthalmic Center, Sun Yat-sen University. All participants provided signed informed consent for their enrollment in this study.

### 2.1. Participants and Sample Selection

**Figure 1:**
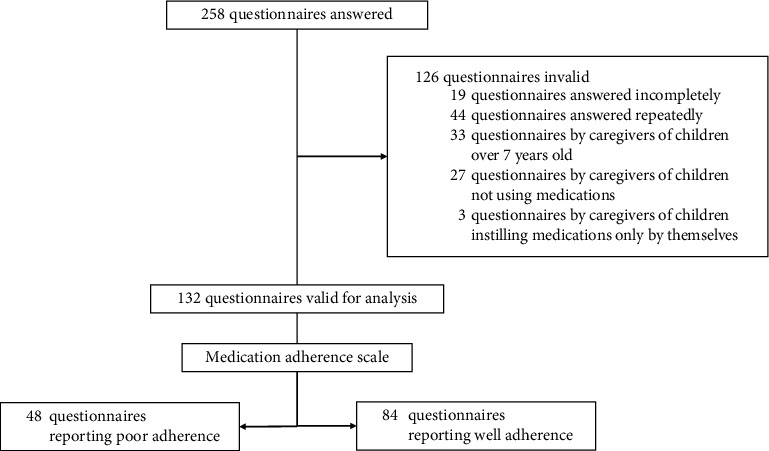
The flow chart showed the number of questionnaires answered, regarded as invalid with specific reasons, and regarded valid for final statistical analysis.

We included caregivers of preschool children with pediatric glaucoma seen at the glaucoma clinic of Zhongshan Ophthalmic Center, Sun Yat-sen University. Only one caregiver of each child from each family was included. Children with pediatric glaucoma were required to fulfill the inclusion criteria: (1) being aged from 0 to 7 years, (2) being diagnosed with pediatric glaucoma (European Glaucoma Society Terminology and Guidelines for Glaucoma, 5th Edition), (3) being taking one or multiple topical antiglaucoma medications (World Glaucoma Association Consensus Series), and (4) having medications instilled only by caregivers. The recruitment took place from January 2019 through October 2021. Trained staff members would help caregivers to answer the questionnaire if needed, including looking up the medical records. Two hundred fifty-eight questionnaires were collected, and 132 (51.2%) of all were considered valid for statistical analysis (Figure 1).

### 2.2. Questionnaire

The questionnaire consisted of 40 items and was divided into 4 sections: (1) demographic information of caregivers, (2) demographic and disease characteristics of children, (3) caregivers-reported antiglaucoma medication adherence, and (4) barriers to medication adherence (Supplement 1).

1. The demographic information of caregivers included age, gender, marital status, employment status, monthly household income, education level, residence, self-evaluated health status, relationship with children, the average number of caregivers for each child in the family, and self-evaluated level of knowledge about pediatric glaucoma in a broad sense.
2. Children's demographic and diseases characteristics consisted of age, gender, type of glaucoma, length of time with a diagnosis of pediatric glaucoma, history of antiglaucoma surgery, length of time with antiglaucoma medication treatment, number of varieties of medications (identified by the bottle), and frequency of medication usage each day.
3. An adapted Morisky Adherence Scale was used to measure glaucoma medication adherence [21, 22]. As most caregivers could not understand English, the questionnaire was asked in their native language. It concludes with 8 items, with a score of 1 being given for the questions answered as NO, and 0 for the questions answered as YES, except for items 2 and 8. The full scale has a range of 0 to 8. Caregivers were categorized as nonadherent when the total score was lower than 6, or they will be regarded as adherent if the total score was 6 or higher.
4. Caregivers were asked to rate the significance of 13 possible barriers to medication adherence via a visual analog scale, which had 11 hatch marks anchored between “strongly disagree and strongly agree.” Then, caregivers ranked the top 3 barriers in sequence among 12 barriers except for anxiety and depression. All 13 barriers were summarized in Table 1.

The 13 barriers were chosen for inclusion after the literature review. In consideration of that, there were not adequate studies concerning medication adherence of pediatric glaucoma, and that adherence greatly depended on caregivers, we included these barriers with reference to studies targeting to caregivers of children with other chronic diseases that required long-term medication treatments, as well as researches targeting to adults with glaucoma.

### 2.3. Statistical Analyses

Means and standard deviations (SD) were used to describe continuous variables, with frequencies and percentages to describe categorical variables. The caregivers would be classified into the nonadherent group if the Morisky adherence score was lower than 6. Independent *t*-test, Chi-square test, and Rank sum test were used to compare the characteristics of children and their caregivers between the adherent and nonadherent groups. Because most barriers were cited as either strongly agrees or strongly disagree, barriers were defined as important if it was rated at the midpoint or more on the visual analog scale. Univariate logistic regression analysis was used to evaluate the association between 13 barriers and poor adherence. And, multiple logistic regression analysis was then conducted to assess the association between barriers statistically significant in univariate logistic regression and poor adherence. Because the caregiver's age and knowledge about pediatric glaucoma were variables associated significantly with medication adherence, we also ran univariate and multiple logistic regression analyses to evaluate the association between the 13 barriers and poor adherence after adjusting for these two variables. In the ranking of barriers, the barrier would score 3 if it was ranked first, score 2 if ranked second, score 1 if ranked third, and score 0 if not ranked. The total scores of each barrier were then ranked. All statistical analyses were performed by SPSS software version 22.0 (IBM SPSS, Armonk, NY, USA). Graphical representation was performed using GraphPad Prism 6.01 (GraphPad Software Inc., USA).

## 3. Results

### 3.1. Participant Characteristics

Forty-eight (36.4%) of 132 children and their caregivers were divided into the nonadherent group. Nonadherent caregivers had a significantly lower mean age than adherent caregivers (30.5 ± 4.1 yrs versus 33.2 ± 5.7 yrs, *P*=0.0498), with a significantly lower level of self-evaluated knowledge about pediatric glaucoma (*P*=0.037). Other demographics of caregivers, as well as demographic and clinical characteristics of children, showed no significant difference between the 2 groups (*P* > 0.05 for all comparisons; Table 2).

Most (82.9%) caregivers were female. Parents consisted of 98.5% of caregivers, with an average number of caregivers for each child being 1.3 ± 1.0 in each family. One hundred twenty-four (93.9%) of all caregivers had a spouse, with 55.3% living in urban areas. Most (93.2%) caregivers had received education for more than six years, with 53.0% working full time and 21.2% working part-time. The largest proportion (27.3%) of them had a monthly household income of more than 1,095.6 dollars. Most (97.0%) of them reported fair to very good health.

Overall, the mean age of included preschool children with pediatric glaucoma was 33.0 ± 21.8 months, and 63.6% of them are male. Primary pediatric glaucoma accounted for 75.8%. The mean length of time with the diagnosis of pediatric glaucoma was 24.0 ± 20.9 months, with 82.6% of them having undergone antiglaucoma surgery treatments. The mean length of time with antiglaucoma medication treatment was 15.9 ± 17.1 months, with 77.3% using multiple glaucoma medications and 97% using medications multiple times per day.

### 3.2. Barriers to Medication Adherence among Nonadherent Group

**Figure 2:**
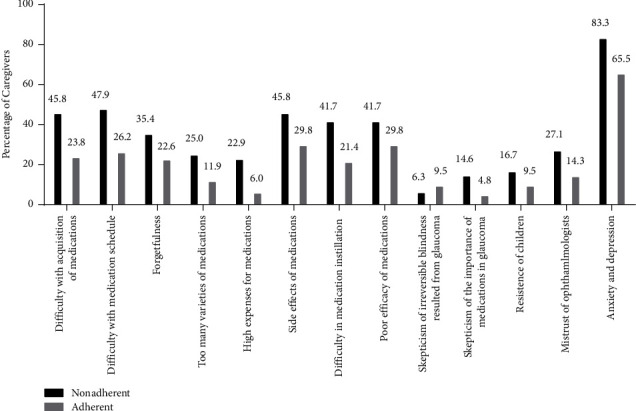
Bar graph showed the percentage of adherent and nonadherent caregivers who rated a barrier at the midpoint or more on the visual analogue scale.

**Figure 3:**
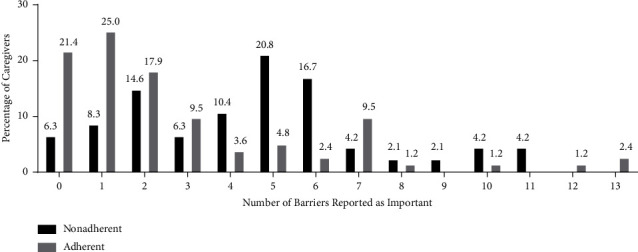
Bar graph showed the frequency of a number of barriers based on the percentage of caregivers, both adherent and nonadherent, who rated different numbers of barriers as important.

In the nonadherent group, all barriers were evaluated as important by 3 (6.3%) or more caregivers. Anxiety and depression (83.3% cited as important), difficulty with medication schedule (47.9% cited as important), difficulty with acquisition of medications (45.8% cited as important), and resistance of children (45.8% cited as important) were most prevalent barriers (Figure 2). Three (6.3%) of caregivers in the nonadherent group cited no barriers as important, 4 (8.3%) cited only 1 barrier, and 41 (85.4%) reported that they encountered multiple barriers simultaneously (Figure 3).

### 3.3. Barriers to Medication Adherence among Adherent Group

For caregivers in the adherent group, each of the 13 barriers was evaluated as important by at least 4 (4.8%) of all. The most common barriers were anxiety and depression (65.5% cited as important), poor efficacy of medications (29.8% cited as important), and resistance of children (29.8% cited as important) (Figure 2). Eighteen (21.4%) of adherent caregivers reported that they had no barriers, 21 (25.0%) reported 1 barrier, and 53 (53.6%) had multiple barriers (Figure 3).

### 3.4. Comparison of Barriers between Nonadherent and Adherent Group

Twenty-one (15.9%) of all caregivers reported no barriers, with 25 (18.9%) reporting 1 barrier and 86 (65.2%) reporting multiple barriers.

Univariate logistic regression analysis indicated that the following barriers were significantly associated with higher odds of poor adherence: difficulty with acquisition of medications (odds ratio (OR), 2.7; 95% confidence interval (CI), 1.3–5.8; *P*=0.010), difficulty with medication schedule (OR, 2.6; 95% CI, 1.2–5.5; *P*=0.012), high expenses for medications (OR, 4.7; 95% CI, 1.5–14.5; *P*=0.007), difficulty with medication instillation (OR, 2.6; 95% CI, 1.2–5.7; *P*=0.015), and anxiety and depression (OR, 2.6; 95% CI, 1.1–6.4; *P*=0.031). For each additional barrier taken as important, there was a 21.3% increased odds of being nonadherent (OR, 1.2; 95% CI, 1.1–1.4; *P*=0.002) (Table 3).

Multiple logistic regression revealed that none of the 5 mentioned significant barriers were still significantly associated with poor adherence (*P* > 0.05 for all).

After adjustment for caregivers' age and knowledge about pediatric glaucoma, univariate logistic regression analysis indicated that difficulty with the acquisition of medications, difficulty with the medication schedule, and high expenses for medications were significantly associated with nonadherence (*P* < 0.05 for all). For each additional barrier cited as important, there was a 17.5% increased odds of being nonadherent (OR, 1.2; 95% CI, 1.0–1.3; *P*=0.016).

After adjusting for age and knowledge about pediatric glaucoma of caregivers, multiple logistic regression revealed that no barriers were significantly associated with poor adherence (*P* > 0.05 for all).

### 3.5. Ranking List of Barriers

**Figure 4:**
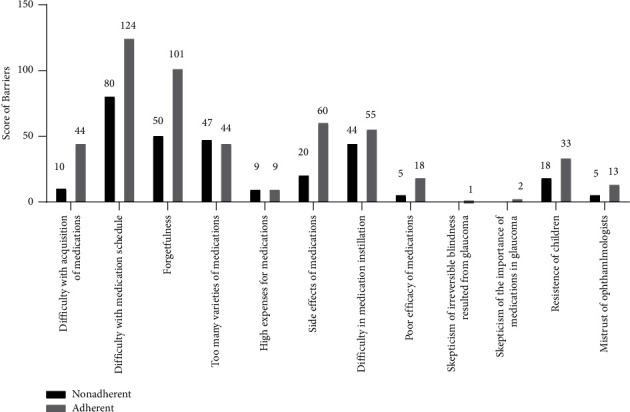
Bar graph showed score of the barriers to medication adherence.

Caregivers were asked to rank their top 3 barriers to medication adherence in sequence according to the degree of severity. Difficulty with the medication schedule, forgetfulness, and too many varieties of medications were the top three barriers listed by caregivers of poor adherence. Difficulty with the medication schedule, forgetfulness, and side effects of medications were cited as the top three barriers by caregivers in the adherent group (Figure 4).

## 4. Discussion

This study found that more than one-third of caregivers of preschool children with pediatric glaucoma were in poor medication adherence administration. Nearly two-thirds of overall caregivers reported that they had multiple barriers to adherence, and each of them had their own unique set of barriers. The more barriers reported as important, the more likely caregivers became nonadherent. Depression and anxiety, difficulty with the medication schedule, difficulty with acquisition of medications, and high expenses for medications were prominent barriers to poor adherence.

In our study, 36.4% of all caregivers reported poor antiglaucoma medication adherence, which is fairly in accordance with a previous survey researching medication adherence in adults with glaucoma [15]. Freedman et al. reported a much higher good adherence in pediatric glaucoma patients (93%), which may result from the specific medication event monitoring system for caregivers used in that study [18]. Electronical techniques could monitor medication instillation accurately and objectively, but they also may remind children and their caregivers to instill medications subconsciously. Moore et al. concluded that caregivers' assessment of medication adherence of their children corresponded well with the electronic monitor if caregivers were responsible for medication administration [38]. Therefore, we think the incidence of poor adherence was credible but still could be undervalued because caregivers may report good adherence due to intentional or unintentional concealment [39, 40].

Our results revealed that lower age and poorer self-evaluated knowledge were the characteristics of nonadherent caregivers. Same as that reported in Newman–Casey's study, higher age was a protective factor for medication adherence [15]. The age of caregivers may interact with other factors, particularly the age of children and the length of time with medication treatment. These variables, however, showed no significant difference between the adherent and nonadherent groups in our study. Limited knowledge is often considered an important obstacle to adherence in glaucoma. Freedman et al. concluded that children of parents with poor health literacy were vulnerable to poor medication administration [18]. Our study also found that nonadherent pediatric glaucoma caregivers tend to have poorer self-evaluated knowledge. Thus, self-assessment of glaucoma knowledge for pediatric glaucoma caregivers may be important, and publicity or education could be provided by network technology, like videos, online clinics, or social media platforms [32, 41–44].

Recent studies found that caregivers of pediatric glaucoma children are experiencing a great caregiving burden due to the required lifelong followup visits and treatments, potential disease progression for their children, loss of wages and bonuses, and children's resistance to medication instillation [35, 45, 46]. Anxiety, depression, and declined quality of life often follow, which may indirectly influence the quality of care they provide, including medication adherence [37, 45, 46]. In our study, a large proportion of caregivers considered anxiety and depression as one of their barriers to optimal adherence, which was in accordance with previous studies focusing on other pediatric diseases like asthma [20, 47]. One potential explanation is that the cognitive impairment caused by depressive emotions, including compromised memory and executive functioning, may contribute to an anxious and depressed caregiver's poor medication adherence administration for their children. Notably, a higher proportion of nonadherent caregivers chose negative emotions as an important barrier when compared with that of the adherent group. This could be explained by the finding that depressive caregivers tend to reflect more problems with disease management [48]. Given these findings, treating caregivers' anxiety and depression may act as a crucial means of improving pediatric glaucoma control. Some brief, validated tools for screening depressive symptoms, as well as social supports and psychological guidance, should be promoted to disseminate negative emotion for pediatric glaucoma caregivers [20].

It is noted that the medication schedule was considered as a crucial barrier to medication adherence for both adherent and nonadherent groups. Unlike adult patients, juggling the schedule of children and caregivers is a common and understandable contributor to nonadherence in pediatric diseases [41], but it is not paid much attention in the management of pediatric glaucoma. In our study, most caregivers were parents, and more than half of them worked full time, which meant that they tend to have a hectic life, with multiple responsibilities in the family and workplace. Even when caregivers understand the prescribed regimen and desire to give their children's medications as required, they usually still have trouble complying because of their hectic schedule or not prioritizing glaucoma medication. It might be necessary for other caregivers in their family, day-care centers, or school (e.g., grandparents, babysitters, and teachers) to share the medication administration responsibility. Moreover, as for children, they may need to be encouraged and taught to instill medications independently at early ages [29, 32].

Certain significant barriers could be coped with by health care workers. In our study, nonadherent caregivers were more likely to be suspicious of the efficacy of medications to adequately lower IOP but bring side effects. In addition, mistrust of ophthalmologists was more salient among nonadherent caregivers, which should raise enough consideration for health care workers. Both physicians and nurses should have a more effective conversation with caregivers and their children. The irreversible damages of pediatric glaucoma and the importance of medication should be strengthened and clarified, which was also consistent with above-mentioned enrichment of knowledge about pediatric glaucoma. In the study of Sleath et al., outcomes expectations were significantly associated with higher adherence in youth with asthma [28]. Therefore, health care workers need to dispel caregivers' worries about the side effects of medications, persuade them into regular and appropriate medication administration, and provide education about outcomes they should expect [41]. Carpenter et al. once reported that higher medication self-efficacy and confidence of patients that they could perform medication-related behaviors well were associated with better adherence [49]. Therefore, it is reasonable that correct operation specifications should be educated for caregivers who took medication instillation tough. Also, children should be taught how to coordinate properly [32]. Medical departments or hospitals, and clinics needed to provide more convenient approaches for caregivers to acquiring medications and to facilitating regular followup so that medication regimens could be adjusted timely.

Some important barriers like high expenses for medications may not be easily resolved in the near future. Although expenses for medications were not as high as those for surgeries and examinations among children with pediatric glaucoma [50]. Our study indicated that the expenses for medications could influence medication adherence significantly. A cost-utility analysis demonstrated that it was beneficial for society to invest in ensuring support for glaucoma patients aged above 40 years to optimize their medication adherence [51]. Therefore, to improve medication adherence may also entail economic support from society.

In this study, caregivers varied in choosing which barriers were important to adherence. Also, the more barriers reported as important, the larger the possibility for caregivers to become nonadherent, which was in accordance with the previous study [15]. It highlighted the necessity for the assessment of adherence and the possible barriers to optimal adherence for each caregiver in each followup. Individualized education or assistance should be provided according to caregivers' own reported barriers, as well as those common barriers encountered by most caregivers [52]. Although a number of glaucoma education programs targeting at different barriers have been developed for adult patients, few programs have been directed at preschool-age children and their families. Future studies and clinical practice should focus more on the development and effectiveness of specific education for children with pediatric glaucoma and their caregivers.

There are several limitations in our study. First, this sample population was from a single one ophthalmic center, so the results may not be representative of children and their caregivers in other ophthalmic centers. Also, some nonadherent caregivers may refuse to participate in this survey in case of exposure of their poor adherence. The ability of physicians to identify participants who were truly nonadherent from their self-reports is also poor [53]. Not only, as some medical records of children might have been lost, recall bias cannot be avoided in this study. A multicenter study was needed in the future to settle or cut down some issues. This study has included a wide range of possible barriers to good adherence based on previous glaucoma and other chronic pediatric diseases studies, but the specific influence of some barriers (e.g., anxiety and depression, medication schedule) on medication adherence in pediatric glaucoma deserves further investigation. In addition, we did not analyze some impediments like race and ethnicity, insurance coverage, as well as depressive symptoms of children, which were previously proven to influence followup adherence for children with pediatric glaucoma or medication adherence of children with asthma [19, 54, 55].

In conclusion, this study demonstrated that more than one-third of caregivers of preschool children with pediatric glaucoma suffered from poor antiglaucoma medication adherence. Anxiety and depression, difficulty with the medication schedule, difficulty with the acquisition of medications, and high expenses for medications were salient barriers to adherence. However, each pair of caregivers and children are likely to have their unique set of barriers, with that the more barriers being reported, the more nonadherent they would be. Hence, individualized assessment and interventions should be provided according to reported barriers by each caregiver.

## Figures and Tables

**Table 1:** Analyzed barriers to medication adherence of children with pediatric glaucoma.

Barriers to medication adherence	Literature sources
Difficulty with acquisition of medications	Tsai et al. (2003) [23], Killeen et al. (2020) [24], Sleath et al. (2009) [25]
Difficulty with medication schedule	Newman-Casey et al. (2015) [15], Lacey et al. (2009) [26]
Forgetfulness	Newman-Casey et al. (2015) [15], Taylor et al. (2002) [27], Sleath et al. (2018) [28]
Too many varieties of medications	Tsai et al. (2003) [23], Committee on school health [29]
High expenses for medications	Tsai et al. (2003) [23], Taylor et al. (2002) [27], Friedman et al. (2008) [30]
Side effects of medications	Taylor et al. (2002) [27], Friedman et al. (2008) [30], Alezzandrini et al. (2014) [31]
Difficulty with medication instillation	McClelland et al. (2019) [16], Taylor et al. (2002) [27]
Poor efficacy of medications	Lacey et al. (2009) [26], Friedman et al. (2008) [30], Gardiner and Dvorkin (2006) [32]
Skepticism of irreversible blindness resulted from glaucoma	Lacey et al. (2009) [26], Friedman et al. (2008) [30]
Skepticism of the importance of medications in treating glaucoma	Tsai et al. (2003) [23], Sanchez et al. (2020) [33]
Resistance of children	Tsai et al. (2003) [23], Sujuan et al. (2015) [34], Law et al. (2020) [35]
Mistrust of ophthalmologists	Tsai et al. (2003) [23], Sleath et al. (2009) [25], Taylor et al. (2002) [27]
Anxiety and depression	Holló et al. (2009) [17], Penkower et al. (2003) [36], Dada et al. (2013) [37]

**Table 2:** Description of children with pediatric glaucoma and caregivers.

Subjects	Variable	Nonadherent (n = 48)	Adherent (n = 84)	Overall (n = 132)	*P* value
Caregivers					
	Age (yrs) (*n* = 131)	30.5 ± 4.1 (*n* = 48)	33.2 ± 5.7 (*n* = 83)	32.2 ± 5.3 (*n* = 131)	0.0498^*∗*^
	Gender				0.260^†^
	Female	42 (87.5)	67 (79.8)	109 (82.6)	
	Male	6 (12.5)	17 (20.2)	23 (17.4)	
	Marital status				1.000^†^
	Having a spouse	45 (93.8)	79 (94.0)	124 (93.9)	
	Having no spouse	3 (6.3)	5 (6.0)	8 (6.1)	
	Employment status				0.338^‡^
	Full-time job	24 (50.0)	46 (54.8)	70 (53.0)	
	Part-time job	8 (16.7)	20 (23.8)	28 (21.2)	
	Out of job	16 (33.3)	18 (21.4)	34 (25.8)	
	Monthly household income ($)				0.992^‡^
	<156.5	5 (10.4)	8 (9.5)	13 (9.8)	
	156.5∼469.6	8 (16.7)	18 (21.4)	26 (19.7)	
	469.6∼782.6	13 (27.1)	20 (23.8)	33 (25.0)	
	782.6∼1,095.6	10 (20.8)	14 (16.7)	24 (18.2)	
	>1,095.6	12 (25.0)	24 (28.6)	36 (27.3)	
	Education level				0.478^‡^
	≤ Primary school	3 (6.3)	6 (7.1)	9 (6.8)	
	Middle school or high school	23 (47.9)	45 (53.6)	68 (51.5)	
	≥ College	22 (45.8)	33 (39.3)	55 (41.7)	
	Residence				0.869^†^
	Urban areas	27 (56.3)	46 (54.8)	73 (55.3)	
	Suburb areas	21 (43.8)	38 (45.2)	59 (44.7)	
	Overall health status				0.182^‡^
	Very poor	1 (2.1)	1 (1.2)	2 (1.5)	
	Poor	2 (4.2)	0 (0)	2 (1.5)	
	Fair	25 (52.1)	39 (46.4)	64 (48.5)	
	Good	14 (29.2)	31 (36.9)	45 (34.1)	
	Very good	6 (12.5)	13 (15.5)	19 (14.4)	
	Relationship with children				1.000^†^
	Parents	47 (97.9)	83 (98.8)	130 (98.5)	
	Other family members	1 (2.1)	1 (1.2)	2 (1.5)	
	Average number of caregivers for each child	1.2 ± 0.9	1.3 ± 0.8	1.3 ± 1.0	0.801^*∗*^
	Knowledge about pediatric glaucoma				0.037^‡£^
	Very poor	4 (8.3)	7 (8.3)	11 (8.3)	
	Poor	7 (14.6)	5 (6.0)	12 (9.1)	
	Fair	30 (62.5)	49 (58.3)	79 (59.8)	
	Good	7 (14.6)	22 (26.2)	29 (22.0)	
	Very good	0 (0)	1 (1.2)	1 (0.8)	

Children					
	Age (months)	27.6 ± 21.8	36.2 ± 21.3	33.0 ± 21.8	0.972^*∗*^
	Gender				0.094^†^
	Female	13 (27.1)	35 (41.7)	48 (36.4)	
	Male	35 (72.9)	49 (58.3)	84 (63.6)	
	Type of glaucoma				0.065^†^
	Primary glaucoma	32 (66.7)	68 (81.0)	100 (75.8)	
	Secondary glaucoma	16 (33.3)	16 (19.0)	32 (24.2)	
	Length of time with a diagnosis of pediatric glaucoma (months)	21.3 ± 21.6	25.6 ± 20.4	24.0 ± 20.9	0.763^*∗*^
	History of antiglaucoma surgery				0.435^†^
	No	10 (20.8)	13 (15.5)	23 (17.4)	
	Yes	38 (79.2)	71 (84.5)	109 (82.6)	
	Length of time with antiglaucoma medication treatment (mths)	13.7 ± 16.7	17.2 ± 17.3	15.9 ± 17.1	0.319^*∗*^
	Number of varieties of antiglaucoma medications (identified by bottle)				0.994^‡^
	1	11 (22.9)	19 (22.6)	30 (22.7)	
	2	12 (25.0)	20 (23.8)	32 (24.2)	
	3	11 (22.9)	22 (26.2)	33 (25.0)	
	4	14 (29.2)	23 (27.4)	37 (28.0)	
	Frequency of antiglaucoma medication usage each day				0.598^‡^
	1	0 (0)	4 (4.8)	4 (3.0)	
	2	15 (31.3)	30 (35.7)	45 (34.1)	
	3	15 (31.3)	16 (19.0)	31 (23.5)	
	≥4	18 (37.5)	34 (40.5)	52 (39.04)	

Data are mean ± standard deviation or number (%). ^*∗*^Two-sample*t*-test. ^†^Chi-square test. ^‡^Rank sum test. ^£^*P* value: 1-tailed. All comparison were made between adherent group and nonadherent group.

**Table 3:** Barriers to medication adherence in children with pediatric glaucoma.

Barrier	Univariate analysis, odds ratio (95% confidence interval)	*P* value	Multiple analysis^*∗*^, odds ratio (95% confidence interval)	*P* value	Univariate analysis^†^, odds ratio (95% confidence interval)	*P* value	Multiple analysis^*∗*^^†^, odds ratio (95% confidence interval)	*P* value
Difficulty with acquisition of medications	2.708 (1.269–5.778)	0.010	1.652 (0.679–4.020)	0.268	2.533 (1.126–5.697)	0.025	1.671 (0.659–4.236)	0.279
Difficulty with medication schedule	2.593 (1.229–5.470)	0.012	1.260 (0.489–3.243)	0.632	2.252 (1.024–4.953)	0.043	1.514 (0.623–3.678)	0.360
Forgetfulness	1.876 (0.858–4.100)	0.115			1.733 (0.752–3.994)	0.197		
Too many varieties of medications	2.467 (0.974–6.245)	0.057			1.812 (0.666–4.928)	0.244		
High expenses for medications	4.697 (1.522–14.497)	0.007	2.768 (0.827–9.270)	0.099	4.768 (1.432–15.868)	0.011	3.354 (0.944–11.912)	0.061
Side effects of medications	3.415 (0.945–12.341)	0.061			3.488 (0.915–13.299)	0.067		
Difficulty with medication instillation	2.619 (1.206–5.686)	0.015	1.623 (0.659–4.000)	0.292	2.048 (0.887–4.729)	0.093		
Poor efficacy of medications	1.686 (0.804–3.534)	0.167			1.632 (0.735–3.626)	0.229		
Skepticism of irreversible blindness resulted from glaucoma	1.900 (0.663–5.441)	0.232			1.248 (0.408–3.819)	0.697		
Skepticism of the importance of medications in treating glaucoma	0.633 (0.160–2.510)	0.516			0.533 (0.129–2.194)	0.383		
Resistance of children	1.997 (0.957–4.167)	0.065			1.467 (0.667–3.226)	0.340		
Mistrust of ophthalmologists	2.229 (0.922–5.386)	0.075			2.417 (0.939–6.219)	0.067		
Anxiety and depression	2.636 (1.091–6.371)	0.031	1.657 (0.642–4.281)	0.297	1.875 (0.744–4.729)	0.183		
Number of barriers	1.213 (1.072–1.372)	0.002			1.175 (1.030–1.339)	0.016		

^*∗*^Only barriers statistically significant in univariate analysis were analyzed for multiple analysis. ^†^Adjusted for age and knowledge about pediatric glaucoma of caregivers.

## Data Availability

The data used to support the findings of this study are available from the corresponding author upon request.

## Supplementary Materials

Supplementary Materials The supplement was the questionnaire used in this study to investigate the medication adherence among preschool children with pediatric glaucoma and possible barriers to adherence.
