# Supplementary material for: Medication Adherence and Common Barriers for Caregivers of Preschool Children with Pediatric Glaucoma
Source: J Ophthalmol. 2022 Dec 31;2022:6389822. doi: 10.1155/2022/6389822 (PMC10238144; doi:10.1155/2022/6389822)
Supplement: Supplementary Materials — The supplement was the questionnaire used in this study to investigate the medication adherence among preschool children with pediatric glaucoma and possible barriers to adherence. [file 6389822.f1.pdf]

Description: This supplement was the questionnaire used in this study to investigate medication adherence among preschool children with pediatric glaucoma and possible barriers to adherence. The original version is in Chinese and translated into English version for presentation.

A Questionnaire about medication adherence among preschool children with pediatric glaucoma

To assist in investigating medication adherence among children with pediatric glaucoma and to clarify common barriers to good adherence, we invite you to take a few minutes to complete this questionnaire carefully and authentically. Your answers would be greatly valued and also would be kept strictly confidential. You can ask for our assistance if any issues. Thank you very much for your support and cooperation!

Part 1. Demographics of caregivers

1. Your age: ____ years
2. Your gender:
   A. Female B. Male
3. Your marital status:
   A. Having a spouse B. Having no spouse
4. Your employment status:
   A. Full-time job B. Part-time job C. Out of job
5. Your household income (Chinese Yuan):
   A. < 1k B. 1k ~ 3k C. 3k ~ 5k D. 5k ~ 7k E. > 7k
6. Your education level:
   A. ≤ Primary school B. Middle school or high school C. ≥ College
7. You are living in:
   A. Urban areas B. Suburb areas
8. How do you feel about your health:
   A. Very poor (having severe diseases or disabilities that limit normal daily activities)
   B. Poor (having moderate disease or disabilities without limitations on normal daily activities)
   C. Fair (having only mild diseases with good prognosis and no limitations on normal daily activities)
   D. Good (having no known disease but feeling not in the best form)
   E. Very good (having no known disease and feeling in best form)
9. For the child, you are his/her:
   A. Parent B. Other family member
10. How many caregivers are there in your family for each child? ____
11. What do you think of your knowledge about pediatric glaucoma in a broad sense?
   A. Very poor B. Poor C. Fair D. Good E. Very good

Part 2. Demographic and disease characteristics of your child

1. Your child's age: ____ months.
2. Your child's gender:
   A. Female B. Male
3. The length of time with diagnosis of pediatric glaucoma for your child: ____ months.
4. Has your child ever undergone anti-glaucoma surgeries?
   A. No B. Yes
5. The length of time with anti-glaucoma medication treatment for your child: ____ months.
6. How many kinds of anti-glaucoma medication is your child using (identified by bottle):
   A. 1 B. 2 C. 3 D.4
7. How many times does your child receive anti-glaucoma medications every day?
   A. One B. Two C. Three D. ≥4

Part 3. Glaucoma medication adherence (adapted from Morisky Adherence Scale)

1. Have you ever forgotten to instill eye drops for your child?
   A. Yes B. No
2. Were there any days when you did not instill eye drops for your child over the past 1 month?
   A. Yes B. No
3. Have you ever stopped instilling eye drops for your child because he/she felt diseases worse?
   A. Yes B. No
4. When your child traveled or left home for a long time, have you ever forgotten to bring along medications for him/her?
   A. Yes B. No
5. Did your children instill eye drops yesterday?
   A. Yes B. No
6. When you felt like your child's intraocular pressure was under control, have you ever stopped instilling eye drops for him/her?
   A. Yes B. No
7. Do you feel hassled about sticking to the child's treatment plan?
   A. Yes B. No
8. How often do you have difficulty in remembering to instill medications for your child?
   A. Never/rarely B. Once in a while C. Sometimes D. Usually E. All the time

Part 4. Possible barriers to glaucoma medication adherence

What do you think of the following barriers affecting medication usage for your child?

1. Difficulty with the acquisition of medications.

0 1 2 3 4 5 6 7 8 9 10

Strongly disagree — Strongly agree

2. Difficulty with the medication schedule.

0 1 2 3 4 5 6 7 8 9 10

Strongly disagree | Strongly agree

3. Forgetfulness.

0 1 2 3 4 5 6 7 8 9 10

Strongly disagree | Strongly agree

4. Too many varieties of medications.

0 1 2 3 4 5 6 7 8 9 10

Strongly disagree | Strongly agree

5. High expenses for medications.

0 1 2 3 4 5 6 7 8 9 10

Strongly disagree | Strongly agree

6. Side effects of medications.

0 1 2 3 4 5 6 7 8 9 10

Strongly disagree | Strongly agree

7. Difficulty with medication instillation.

0 1 2 3 4 5 6 7 8 9 10

Strongly disagree | Strongly agree

8. Poor efficacy of medications.

0 1 2 3 4 5 6 7 8 9 10

Strongly disagree | Strongly agree

9. Skepticism of irreversible blindness resulting from glaucoma.

0 1 2 3 4 5 6 7 8 9 10

Strongly disagree — Strongly agree

10. Skepticism of the importance of medications in treating glaucoma.

0 1 2 3 4 5 6 7 8 9 10

Strongly disagree — Strongly agree

11. Resistance of children.

0 1 2 3 4 5 6 7 8 9 10

Strongly disagree — Strongly agree

12. Mistrust of ophthalmologists.

0 1 2 3 4 5 6 7 8 9 10

Strongly disagree — Strongly agree

13. Anxiety and depression

0 1 2 3 4 5 6 7 8 9 10

Strongly disagree — Strongly agree

Please rank the top 3 barriers in sequence that you think most affect medication usage.

- ○ Difficulty with the acquisition of medications
- ○ Difficulty with the medication schedules
- ○ Forgetfulness
- ○ Too many varieties of medications
- ○ High expenses for medications
- ○ Side effects of medications
- ○ Difficulty with medications instillation
- ○ Poor efficacy of medications
- ○ Skepticism of irreversible blindness resulting from glaucoma
- ○ Skepticism of the importance of medications in treating glaucoma
- ○ Resistance of children
- ○ Mistrust of ophthalmologists
